# Growing Up With Terrorism: The Age at Which a Terrorist Attack Was Suffered and Emotional Disorders in Adulthood

**DOI:** 10.3389/fpsyg.2021.700845

**Published:** 2021-06-18

**Authors:** Sara Prieto, Jesús Sanz, María Paz García-Vera, Rocío Fausor, Noelia Morán, Beatriz Cobos, Clara Gesteira, Roberto Navarro, Pedro Altungy

**Affiliations:** Department of Personality, Assessment, and Clinical Psychology, Complutense University of Madrid, Madrid, Spain

**Keywords:** post-traumatic stress disorder, anxiety disorders, depressive disorders, terrorism, childhood, adolescence, adulthood, trauma

## Abstract

Abundant scientific literature shows that exposure to traumatic situations during childhood or adolescence has long-term psychopathological consequences, for example, in the form of a higher prevalence of emotional disorders in adulthood. However, an evolutionary perspective suggests that there may be differential vulnerabilities depending on the age at which the trauma was suffered. As there are no studies on the psychopathological impact in adulthood of attacks suffered during childhood or adolescence, the objective of this study was to analyze the influence of the age at which a terrorist attack was suffered in the presence of emotional disorders many years after the attack. A sample of 566 direct and indirect victims of terrorist attacks in Spain was recruited, of whom 50 people were between the age of 3 and 9 when they suffered the attack, 46 were between 10 and 17 years old, and 470 were adults. All of them underwent a structured diagnostic interview (SCID-I-VC) an average of 21 years after the attacks. No significant differences were found between the three age groups at which the attack occurred in terms of the current prevalence of post-traumatic stress disorder, major depressive disorder, or anxiety disorders. The results of several multiple binary logistic regression analyses also indicated that, after controlling for the effect of sex, current age, the type of victims, and the time since the attack, the age at which the attack was suffered was not related to the current prevalence of those emotional disorders. The results are discussed concerning the differences between various types of trauma and in the context of the theories that propose that traumatic experiences are processed differently at different ages and can lead to differences in the likelihood of developing different emotional disorders.

## Introduction

A large number of reviews of the scientific literature have found a high prevalence of symptoms and emotional disorders in children and adolescents who suffered a terrorist attack directly or indirectly (Fremont, 2004; Comer and Kendall, 2007; Neria et al., 2011; Perlman et al., 2011; Pereda, 2013; Slone and Mann, 2016). For example, the prevalence of post-traumatic stress disorder (PTSD) in children and adolescents between 4 and 13 months after suffering a terrorist attack ranges from 10 to 30% (García-Vera et al., 2021; but see Pat-Horenczyk, 2005, for lower prevalence figures).

The scientific literature has also found that exposure to traumatic situations during childhood or adolescence has long-term psychopathological consequences in adulthood. These include increased presence of depressive disorders, anxiety disorders, PTSD, or their symptoms (Maercker et al., 2004; Kessler et al., 2010; Maschi et al., 2013; Copeland et al., 2018), especially in adults who have suffered some form of maltreatment (Li et al., 2016; Nelson et al., 2017) or sexual abuse (Hailes et al., 2019) during their childhood or adolescence.

However, it is unclear whether having suffered a terrorist attack in childhood or adolescence also poses an increased risk of developing emotional disorders in adulthood compared to a terrorist attack that was suffered during adulthood itself.

On the one hand, children and adolescents who experience a terrorist attack directly or indirectly would be expected to present more emotional symptoms and disorders in adulthood than people who were already adults when they experienced a terrorist attack directly or indirectly, for at least three reasons. First, unlike many experiences that minors can undergo during their development, the direct or indirect experience of a terrorist attack impacts all the contexts in which the child is developing (e.g., family, peer group, school, society, social, and communication media) and is therefore expected to have an important effect in many areas of their development (Comer and Kendall, 2007). Second, the direct or indirect experience of a terrorist attack forces minors to face the reality that some human beings wish to harm or even kill them, therefore questioning their trust and beliefs about the goodness of human beings, the safety of the world, or their personal invulnerability precisely at the time such beliefs are developing (Comer and Kendall, 2007). Third, research in the areas of developmental traumatology and developmental psychopathology suggests that childhood experience of a traumatic event, such as a terrorist attack, may compromise the maturation of the mechanisms that regulate emotions or mood (Maercker et al., 2004) or may have negative effects on the development of stress-related biological systems (Heim and Nemeroff, 2001; Comer and Kendall, 2007).

On the other hand, some studies have found that there are differences in the psychological impact that terrorist attacks have on children and adolescents depending on their age. For example, 6 months after the 9–11 attacks, Hoven et al. (2005) found that, among New York City public school children, 9–11 year olds showed higher rates of probable PTSD, probable social anxiety disorder, and probable agoraphobia than 11–18 year-olds. It could therefore also be assumed that, among children and adolescents who have suffered a terrorist attack, the younger ones may have more psychopathological consequences as adults than the older ones.

Another possibility is that differences in the children's age had not so much to do with a higher or lower likelihood of developing general psychological disorders as adults, but with the likelihood of developing to a greater extent only certain psychological disorders as adults. From an evolutionary perspective, traumatic experiences are supposed to be processed differently at different ages and can therefore lead to differences in the likelihood of developing different mental disorders depending on the age of the child when they suffered the attack. For example, suffering an attack in adolescence, compared to suffering it in childhood, could increase vulnerability to PTSD, as the development of PTSD requires some maturation of memory organization and modulation of activation that is not achieved before adolescence (Pynoos et al., 1999). The very nature of intrusive reexperiencing, typical of PTSD, requires the recording, processing, and analysis of sensory information with kinetic and somatic information, which depends on frontal cortical development. More importantly, the development of PTSD requires the presence of certain dysfunctional beliefs about the dangers of the world, human evil, personal vulnerability, a hopeless future, etc., and the development of these beliefs depends on the child's cognitive maturity because, for example, deductive hypothetical thinking, moral and ethical reasoning, or reflection on things that happen to us begin to develop significantly as of adolescence. In this sense, Maercker et al. (2004) found, in a sample of women aged 18–45 years, that those who had suffered a traumatic event during their adolescence, between the age of 13 and 18, were at greater risk of PTSD than of suffering a major depressive disorder (MDD) (13.3 vs. 6.5%), whereas women who had experienced a traumatic event during their childhood, before the age of 12, had the same risk of PTSD as of MDD (17 vs. 23.3%). In this same study, although no significant differences in the risk of PTSD were found between women who had experienced a traumatic event in adolescence and those who had experienced it in childhood, an increased risk of developing MDD was found if the traumatic event had occurred in childhood rather than in adolescence (23.3 vs. 6.5%). This latter finding could be related to the differences between childhood and adolescence regarding the maturation of psychological or psychophysiological mechanisms of mood regulation or stress response (Maercker et al., 2004).

In the same direction, children, and adolescents differ in their ability to mentally represent the future, and this ability could be associated with the degree to which the child can worry about future terrorist attacks (Comer and Kendall, 2007). As a result, compared to children, adolescents may be more likely to suffer from emotional disorders in which worry is a key feature, such as generalized anxiety disorder, or where hypervigilance about future attacks is a key feature, such as PTSD.

In the context of the various above-mentioned lines of research, the objective of this study was to analyze the influence of the age at which a terrorist attack was suffered in the presence of PTSD, depressive disorders, and anxiety disorders many years after the attack. In particular, this study examined, first, whether people who suffered a terrorist attack as children or adolescents showed more emotional disorders as adults than those who suffered a terrorist attack as adults. Second, it examined whether there were differences in the presence of emotional disorders in adulthood between those who were children when the terrorist attack occurred to them and those who were adolescents. Finally, it also examined whether suffering a terrorist attack in childhood can increase both the risk of PTSD and the risk of MDD or anxiety disorders in adulthood whereas suffering an attack in adolescence is more likely to increase the risk of PTSD in adulthood than the risk of MDD or anxiety disorders.

## Methods

### Participants

To carry out this study, a sample of 566 adult victims of terrorism who were members of the Association of Victims of Terrorism (AVT) of Spain was recruited in two phases. In the first phase, 1,587 adults who belonged to the AVT who had been injured in a terrorist attack or were first-degree relatives of a person who was injured or killed in a terrorist attack were contacted by telephone. Of these people, 1,325 participated in a telephone interview and completed various psychopathological questionnaires, while 32 requested to carry out this telephone evaluation in person. In a second phase, an appointment was made with these 1,367 victims to conduct a face-to-face interview that included a structured diagnostic interview to assess the presence of emotional disorders. Of the total number of people invited to participate in the face-to-face interview, 601 completed it and, of these, 566 were 3 years of age or older when they suffered the terrorist attack. These individuals made up the definitive sample of victims of terrorism that participated in this study. We decided to exclude people who were younger than 3 years old when the attack occurred because abundant empirical literature shows that the earliest memories in most people date, at most, from when they were 3–4 years old (Joseph, 2003; Kingo et al., 2013; Akhtar et al., 2018).

The average age of the final sample of 566 participants when they were evaluated was 51.31 years (*SD* = 13.42), the percentage of women was 53.2%, and the average number of years elapsed since the attack to the time they underwent the face-to-face interview was 20.98 years (*SD* =10.04). Of these participants, 40.8% had been injured in a terrorist attack, 36.2% were first-degree relatives of a person who was killed in an attack, and the remaining 23% were first-degree relatives of a person who was injured in an attack.

Based on the definition of adolescence of the World Health Organization (1986), which considers that this stage begins as of the age of 10, the sample of participants was divided into the following three groups according to their age at the time they suffered the attack: between 3 and 9 years (childhood; *n* = 50), between 10 and 17 years old (adolescence; *n* = 46), and 18 years of age or older (adult age; *n* = 470). Table 1 presents the sociodemographic characteristics and the attack-related characteristics of these three groups of participants.

**Table 1:** Sociodemographic characteristics and attack-related characteristics of the groups of participants as a function of their age when they suffered the terrorist attack (3–9, 10–17, or 18 years or older) and significant differences between the groups in these characteristics.

**Characteristic**	**3–9 years**	**10–17 years**	**≥ 18 years**	***p* of **χ**^**2**^/*F***
	**(*n* = 50)**	**(*n* = 46)**	**(*n* = 470)**	
Sex: % of women	56.0	47.8	53.4	0.705
Current mean age in years (*SD*)	33.22_a_ (6.98)	38.17_a_ (9.58)	54.52_b_ (11.87)	0.001
Average time since the attacks in years (*SD*)	26.76_a_ (6.87)	23.43_a, b_ (10.27)	20.13_b_ (10.08)	0.001
Average age at the time of the attack in years (*SD*)	5.98_a_ (1.88)	13.85_b_ (2.14)	33.97_c_ (10.45)	0.001
Injured victim: %	4.0_a_	19.6_a_	46.8_b_	0.001
Relative of the deceased: %	66.0_a_	47.8_a, b_	31.9_b_	0.001
Relative of the injured: %	30.0	32.6	21.3	0.102

*In the case of significant χ^2^ or F-tests, the means or percentages with the same subscripts do not differ significantly at p < 0.05*.

### Variables and Instruments

#### Sociodemographic Characteristics and Attack-Related Characteristics of the Participants

A semi-structured interview was conducted, created *ad-hoc* and based, in part, on the general module of the Structured Clinical Interview for AXIS I Disorders of DSM-IV, clinical version (SCID-I-CV; First et al., 1997), in its Spanish translation (First et al., 1999), and on the trauma interview of Foa et al. (2007).

#### Diagnosis of PTSD, Anxiety, and Depressive Disorders According to the DSM-IV

Modules F (anxiety and other disorders) and A (affective episodes) of the SCID-I-CV (First et al., 1997, 1999) were applied. Diagnostic measures derived from the SCID-I-CV have good indices of validity and reliability (Sanz, 2013). For example, concerning the DSM-IV psychological disorders assessed in the present study, the range of inter-rater reliability indices (kappa) from different studies oscillates from good (>0.70) to fair (0.50–0.70); in particular, the range is 0.61–0.80 for MDD, 0.65–0.67 for panic disorder, 0.63–0.75 for generalized anxiety disorder, and 0.77–0.88 for PTSD (Zanarini et al., 2000; Lobbestael et al., 2011).

### Procedure

During the face-to-face interview, all participants were individually evaluated by psychologists adequately trained in conducting psychological assessments and treatments with victims of terrorism through a continuous formation diploma, observing interviews, and conducting weekly clinical sessions.

After obtaining their informed written consent, during that face-to-face individual evaluation session, all participants underwent the semi-structured interview to collect their sociodemographic and attack-related characteristics, and then, the SCID-I-VC was applied.

### Statistical Analyses

The statistical analyses performed in this study were carried out with the SPSS statistical program for Windows, version 25. The prevalence of PTSD, MDD, panic disorder, specific phobia, generalized anxiety disorder, social phobia, agoraphobia, obsessive-compulsive disorder, any mood disorder, any anxiety disorder, and any emotional disorder was calculated for each of the three groups created based on the age at which the attack was suffered—childhood (3–9 years), adolescence (10–17 years), and adulthood (18 years and older)—. Given the characteristics of the diagnostic interview used in the present study—a face-to-face interview administered by a clinician—, there were no missing values regarding the diagnosis for the participants who finally completed the SCID-I-VC. Differences in prevalence between these three groups were examined with chi-square tests and, if a chi-square test was statistically significant, the prevalence of the three groups was compared in pairs with *z*-tests and the corrected *p*-values through the Bonferroni method. These same analyses were carried out to examine the differences between the three groups in terms of the sociodemographic variables and attack-related variables that were categorical (sex and type of victim), whereas if the variables were continuous (current mean age, average time since the attack, and mean age at the time of the attack), one-way ANOVAs were conducted and if the *F*-tests of these ANOVAs were significant, Bonferroni's *post-hoc* tests were calculated to compare the groups in pairs.

As the differences between the three groups in terms of the prevalence of different emotional disorders could be due to the influence not only of the age at which the attack was suffered, but of other confounding variables, multiple binary logistic regression analyses were carried out on the dichotomous variable presence or absence of each of the emotional disorders, entering the following predictors: sex, current age, type of victim, time since the attack, and the age of the victim at the time of the attack. Of these predictors, type of victim was a qualitative variable with three categories: injured in the attack, relative of someone killed in the attack, and relative of someone injured in the attack. Two dichotomous dummy variables were created for this variable: having been injured (compared to being a relative of someone killed or a relative of someone injured) and being a relative of someone killed (compared to being injured or being a relative of someone injured). Multiple binary logistic regression analyses were conducted only on emotional disorders with frequencies >30, to reduce the risk of finding biased or incongruous results (Ortega Calvo and Cayuela Domínguez, 2002; Vittinghoff and McCulloch, 2007).

## Results

### Differences in Sociodemographic Characteristics and Attack-Related Characteristics Among Participants Who Suffered an Attack in Childhood, Adolescence, or Adulthood

Table 1 presents the results of the statistical tests carried out to examine the differences between the groups of participants who suffered an attack in childhood, adolescence, or adulthood in their sociodemographic characteristics and the characteristics related to the attacks.

As expected, compared to the groups that suffered the attack in childhood or adolescence, the group of participants who suffered the attack in adulthood had a significantly higher current mean age (*p* < 0.001), included a higher percentage of people injured in the attacks (*p* < 0.001), and a lower percentage of relatives of people killed in the attacks (*p* < 0.001), and was assessed a shorter time after suffering the attack (*p* < 0.001) (see Table 1). However, no differences were found between the three groups of participants in the percentage of women or of relatives of those injured in the attacks (*p* < 0.705 and 0.102, respectively), nor were there any differences between the group that suffered the attack in childhood and the one that suffered it in adolescence in terms of their current mean age, the percentage of people injured in the attacks or the relatives of people killed in the attacks or the mean time since the attack (all tests with *p* > 0.05).

### Differences in the Prevalence of Emotional Disorders in Adulthood Among Participants Who Suffered a Terrorist Attack in Childhood, Adolescence, or Adulthood

Table 2 presents the prevalences of the different emotional disorders found in adulthood in the three age-groups of participants as a function of when they suffered the terrorist attack. The results of the statistical tests that were conducted to examine the differences between the three groups in the prevalence of emotional disorders revealed that such differences were not statistically significant for any of the eight individual emotional disorders listed in Table 2 (all tests with *p* > 0.05), except for the case of obsessive-compulsive disorder (*p* <0.046). For this disorder, a higher prevalence was found in participants who had suffered a terrorist attack as 3–9 years olds (10%) than in participants who had suffered it as adults (4.1%), or who had suffered it when they were adolescents between ages 10–17 years (0%). However, although the overall chi-square test revealed that such differences were significant, subsequent tests comparing the three groups in pairs failed to reveal any statistically significant differences.

**Table 2:** Prevalence (%) of emotional disorders in victim groups based on their age when they suffered the terrorist attack (3–9, 10–17, or 18 years or older) and significant differences between the groups in that prevalence.

**Emotional disorder**	**3–9 years**	**10–17 years**	**≥ 18 years**	***p* of **χ**^**2**^**
	**(*n* = 50)**	**(*n* = 46)**	**(*n* = 470)**	
Posttraumatic stress disorder	22.0	30.4	26.2	0.643
Major depressive disorder	14.3	13.0	19.8	0.376
Panic disorder	12.2	15.2	12.6	0.870
Specific phobia	12.2	10.9	14.9	0.685
Generalized anxiety disorder	6.0	13.0	10.7	0.494
Social phobia	2.0	6.5	5.8	0.513
Agoraphobia	0.0	2.2	5.5	0.151
Obsessive-compulsive disorder	10.0_a_	0.0_a_	4.1_a_	0.046
Any mood disorder	16.0	26.1	24.7	0.368
Any anxiety disorder	36.0	37.0	38.2	0.947
Any emotional disorder	42.0	47.8	52.1	0.360

*In the case of significant χ^2^ tests, the percentages with the same subscripts do not differ significantly at p < 0.05*.

The small size of the groups of participants who had suffered the attack as children or adolescents (*n* = 50 and 46, respectively), coupled with the low prevalence of obsessive-compulsive disorder (between 0 and 10%) and other emotional disorders (e.g., social phobia: 2–6%; agoraphobia: 0–5%) could explain the absence of statistically significant differences between the three groups of participants not only regarding the presence of obsessive-compulsive disorder but also concerning those other emotional disorders. However, it is important to note that, in the case of the most common emotional disorders, such as PTSD, MDD, or panic disorder, or the case of groups of emotional disorders (any mood disorder, any anxiety disorder, or any emotional disorder), no statistically significant differences were found in their prevalence. However, for example, in the case of PTSD, the prevalence figures ranged from 22 to 30% and, in the case of the presence of any emotional disorder, the figures ranged from 42 to 52%.

As the degree of exposure to terrorist attacks is the variable most strongly related to the risk of emotional disorders arising from attacks and the persistence of such disorders (Perlman et al., 2011; Pereda, 2013; García-Vera et al., 2021), the absence of statistically significant differences in the prevalence of emotional disorders depending on the age at which the attack was suffered may be related to the fact that a significant part of the sample of participants were indirect victims of the attacks-−59.2% of the participants were relatives of those killed or injured in attacks—. Table 3 shows the prevalence of different emotional disorders among participants who were direct victims of terrorism based on their age when they were injured in a terrorist attack, although, in this case, only two age groups, childhood-adolescents vs. adults, were taken into account, given the small number of participants who had been injured in an attack as children or adolescents. The results of the statistical analyses confirmed the results obtained with the complete sample of participants, because, considering only the direct victims of terrorism, no statistically significant differences were found between the participants who had been injured in an attack when they were 3–17 years old and those who had been injured when they were adults for any of the eight individual emotional disorders listed in Table 3 or for any of the three groups of emotional disorders listed in this table (all tests with *p* > 0.05).

**Table 3:** Prevalence (%) of emotional disorders in groups of those injured in attacks based on their age when they suffered the terrorist attack (3–17 or 18 years or older) and significant differences between the groups in that prevalence.

**Emotional disorder**	**3–17 years**	**≥ 18 years**	***p* of **χ**^**2**^**
	**(*n* = 11)**	**(*n* = 220)**	
Posttraumatic stress disorder	45.5	35.0	0.479
Major depressive disorder	9.1	22.7	0.287
Panic disorder	18.2	13.6	0.670
Specific phobia	9.1	14.5	0.614
Generalized anxiety disorder	9.1	10.9	0.850
Social phobia	0.0	6.8	0.370
Agoraphobia	0.0	5.5	0.426
Obsessive-compulsive disorder	9.1	4.5	0.490
Any mood disorder	18.2	27.3	0.507
Any anxiety disorder	36.4	39.5	0.833
Any emotional disorder	63.6	56.8	0.656

On another hand, it is difficult to establish an age to distinguish between childhood, adolescence, and adulthood, because age is actually a simplistic approach to people's level of cognitive, social, or emotional development (Rutter, 1989). Therefore, the absence of statistically significant differences in the prevalence of emotional disorders may have to do with the ages used to create the participants' age groups when the terrorist attack occurred. In previous studies on the influence of traumatic experiences during childhood or adolescence in adult psychopathology, 13 years of age has been used to distinguish between childhood and adolescence (Maercker et al., 2004). As a result, the prevalence of different emotional disorders was also calculated for participants who suffered the attack when they were between the ages of 3 and 12, when they were between the ages of 13 and 17, and when they were 18 years of age or older (see Table 4). The results of statistical analyses comparing the prevalence of emotional disorders in these three new groups were similar to those obtained with the original three groups; that is, no significant difference was found in the prevalence of PTSD, MDD, or any of the anxiety disorders or the prevalence of emotional disorder clusters (all tests with *p* > 0.05; see Table 4).

**Table 4:** Prevalence (%) of emotional disorders in groups of participants based on their age when they suffered a terrorist attack (3–12, 13–17, or 18 years or older) and significant differences between groups in that prevalence.

**Emotional disorder**	**3–12 years**	**13–17 years**	**≥ 18 years**	***p* of **χ**^**2**^**
	**(*n* = 65)**	**(*n* = 31)**	**(*n* = 470)**	
Posttraumatic stress disorder	24.6	29.0	26.2	0.899
Major depressive disorder	15.6	9.7	19.8	0.299
Panic disorder	14.1	12.9	12.6	0.944
Specific phobia	10.9	12.9	14.9	0.675
Generalized anxiety disorder	6.2	16.1	10.7	0.305
Social phobia	3.1	6.5	5.8	0.654
Agoraphobia	0.0	3.2	5.5	0.134
Obsessive-compulsive disorder	7.7	0.0	4.1	0.191
Any mood disorder	20.0	22.6	24.7	0.691
Any anxiety disorder	35.4	38.7	38.2	0.906
Any emotional disorder	43.1	48.4	52.1	0.377

Finally, the results of statistical analyses examining differences in the prevalence of emotional disorders in adulthood among participants who suffered an attack in childhood, adolescence, or adulthood were also the same when the final sample of participants in this study included the 35 participants who had initially been excluded for having suffered the terrorist attack before the age of 3. These cases were excluded because it is unlikely that a person of that age has any memory of any event (Joseph, 2003; Kingo et al., 2013; Akhtar et al., 2018). However, when estimating the psychopathological consequences of a terrorist attack, it is necessary to analyze the characteristics of the terrorist attack itself, but also the contexts of violence and threat in which it occurred, as well as the political, social, and cultural characteristics of the community affected by the attack (García-Vera et al., 2021). These characteristics and those contextual variables may last many years after the terrorist attack and, therefore, they may affect to victims even 3 years after the terrorist attack. For example, victims of terrorism in Spain, especially in the Spanish autonomous community of the Basque Country, compared to the victims of the attacks that occurred, for example, in the USA, have experienced intense and repeated direct or indirect exposure to terrorist attacks during many years and have subsequently suffered many stressful events related to them (e.g., street violence related to terrorism; continued personal threats by terrorists or their environment; contempt, humiliation, rejection, and stigmatization by people close to the terrorist environment) (García-Vera et al., 2021; Sanz and García-Vera, 2021). For this reason, it is relevant to repeat the statistical analysis of the participant sample with the 35 participants who had initially been excluded for having suffered the terrorist attack before the age of 3 years. However, for the sake of brevity, the results of the latter analyses are presented in the Supplementary Material.

### Multiple Binary Logistic Regression Analyses on the Prevalence of Emotional Disorders in Adulthood

Table 5 presents the results of the multiple binary logistic regression analyses on the presence in adulthood of various emotional disorders. These results revealed that age at the time of the attack was not significantly associated with the presence in adulthood of PTSD, MDD, panic disorder, specific phobia, any mood disorder, any anxiety disorder, or any emotional disorder after controlling the effects of sex, current age, the fact of being injured in an attack, the fact of being a relative of a person who was killed in an attack, and the time since the attack (see Table 5).

**Table 5:** Results of the multiple binary logistic regression analyses on the presence of emotional disorders in the sample of victims of terrorism.

**Emotional disorders/Predictors**	**B**	***p***	**Exp(B): *OR***
**Posttraumatic stress disorder**			
**Sex**	**0.886**	**0.001**	**2.42**
Current age	−0.002	0.927	0.99
**Injured**	**1.462**	**0.001**	**4.31**
Relative of the deceased	0.504	0.082	1.66
Time since the attack	−0.002	0.311	0.99
Age at the time of the attack	−0.016	0.512	0.98
**Major depressive disorder**			
**Sex**	**1.155**	**0.001**	**3.17**
Current age	0.003	0.913	1.00
**Injured**	**1.032**	**0.001**	**2.81**
Relative of the deceased	0.419	0.185	1.52
Time since the attack	−0.002	0.504	0.99
Age at the time of the attack	−0.006	0.819	0.99
**Panic disorder**			
**Sex**	**1.190**	**0.001**	**3.29**
Current age	−0.005	0.871	0.995
**Injured**	**0.803**	**0.024**	**2.32**
Relative of the deceased	0.291	0.406	1.34
Time since the attack	−0.002	0.509	0.99
Age at the time of the attack	−0.022	0.430	0.98
**Specific phobia**			
**Sex**	**0.546**	**0.035**	**1.73**
Current age	−0.003	0.930	0.99
Injured	0.171	0.591	1.19
Relative of the deceased	0.268	0.403	1.31
Time since the attack	−0.004	0.161	0.99
Age at the time of the attack	−0.013	0.662	0.99
**Any mood disorder**			
**Sex**	**0.976**	**0.001**	**2.65**
Current age	−0.002	0.929	0.99
**Injured**	**0.988**	**0.001**	**2.69**
**Relative of the deceased**	**0.577**	**0.043**	**1.78**
Time since the attack	−0.001	0.580	0.99
Age at the time of the attack	−0.004	0.879	0.99
**Any anxiety disorder**			
**Sex**	**0.930**	**0.001**	**2.53**
Current age	0.009	0.701	1.01
**Injured**	**0.706**	**0.004**	**2.03**
**Relative of the deceased**	**0.499**	**0.037**	**1.65**
**Time since the attack**	**−0.004**	**0.047**	**0.99**
Age at the time of the attack	−0.032	0.172	0.97
**Any emotional disorder**			
**Sex**	**1.039**	**0.001**	**2.82**
Current age	0.011	0.640	1.01
**Injured**	**1.125**	**0.001**	**3.08**
**Relative of the deceased**	**0.524**	**0.024**	**1.69**
Time since the attack	−0.004	0.066	0.99
Age at the time of the attack	−0.028	0.240	0.97

*Statistically significant predictors appear in bold*.

In fact, the only variables that were consistently and significantly related to the presence of emotional disorders in adulthood were sex and the fact that the respondent was injured in an attack, such that women and the victims injured in an attack showed an increased risk of PTSD, MDD, panic disorder, any mood disorder, any anxiety disorder, or any emotional disorder (all significant regression coefficients with *p* < 0.05). For example, female victims had more than twice the risk of PTSD than male victims (*OR* = 2.42), whereas the victims injured in an attack had more than four times the risk of PTSD than victims who were relatives of people injured or killed in attacks (*OR* = 4.31). Moreover, in the case of the presence of any mood disorder, any anxiety disorder, and any emotional disorder, the fact of being a relative of a person killed in an attack also appeared as a statistically significant predictor, such that victims who were relatives of a person killed in an attack showed an increased risk of developing emotional disorders in general (*OR* between 1.55 and 1.78).

The influence of age at the time of the attack on the presence of mental disorders in adulthood may be limited to childhood and adolescence, not covering adulthood, such that the null relationship in this period may conceal the relationship that does appear in childhood and adolescence, especially when most of the sample of participants (83%) had suffered the attack as adults. Therefore, the multiple binary logistic regression analyses were repeated only with participants who had suffered the attack as children or adolescents. The results of these analyses, which for the sake of brevity are presented in the Supplementary Material, were the same concerning the influence of age at the time of the attack. In particular, once the other predictors were controlled, age at the time of the attack, again, was not significantly associated with the presence in adulthood of PTSD, MDD, panic disorder, specific phobia, any mood disorder, any anxiety disorder, or any emotional disorder.

Finally, the results of the multiple binary logistic regression analyses were also the same concerning the influence of age at the time of the attack when the final sample of participants in this study included the 35 participants who had initially been excluded for having suffered the terrorist attack before the age of 3. For the sake of brevity, the results of the latter analyses are also presented in the Supplementary Material.

## Discussion

There is abundant empirical literature that has examined the relationship between exposure to different traumatic situations during childhood or adolescence and the presence of emotional symptoms and disorders in adulthood (Maercker et al., 2004; Kessler et al., 2010; Maschi et al., 2013; Nelson et al., 2017; Copeland et al., 2018; Hailes et al., 2019). However, to our knowledge, this is the first study that addresses this issue specifically in people who have suffered a terrorist attack and, in particular, that addresses the influence of the age at which a terrorist attack was suffered on the presence of PTSD, depressive disorders, and anxiety disorders many years after the attack.

The results of the study indicate, first, that people who have suffered a terrorist attack as children or adolescents do not present more emotional disorders as adults than those who have suffered a terrorist attack as adults. In the Introduction to this work, we presented a number of theoretical arguments that would justify the higher long-term psychological impact that terrorist attacks would have when they occur in childhood or adolescence, as they would supposedly affect the development of many psychological, biological, and social areas of the individual (e.g., mechanisms of emotional regulation, biological systems related to stress response, and formation of beliefs about the goodness of human beings or the safety of the world, among others) (Maercker et al., 2004; Comer and Kendall, 2007). Despite these arguments, neither the comparison of the prevalence of emotional disorders nor the multiple binary logistic regression analyses of this prevalence performed in this study revealed results that support the idea that the age at which a terrorist attack was suffered is significantly associated with its psychopathological consequences in adulthood. Moreover, in this study, we found no significant differences or relationships between the psychopathological consequences in adulthood and the age at which the terrorist attack was suffered, which was consistent in various emotional disorders, namely PTSD, MDD, panic disorder, specific phobia, generalized anxiety disorder, social phobia, agoraphobia, and obsessive-compulsive disorder.

A second finding of this study is that there are no differences in the prevalence of emotional disorders in adulthood between people who suffered a terrorist attack as children and those who suffered it as adolescents. This finding is consistent with the results of Maercker et al. (2004) who found no significant differences in the risk of PTSD between women who had experienced a traumatic event in adolescence and those who had experienced it in childhood. However, this study found an increased risk of developing MDD if the traumatic event had occurred in childhood than if it had occurred in adolescence, whereas in our study, there were no differences in the risk of MDD, nor were there any differences in the risk of PTSD or different anxiety disorders.

In fact, a third finding of this study is that there are no differences between people who suffered a terrorist attack as children and those who suffered it as adolescents in terms of a differential pattern of the presence of emotional disorders. For example, in both groups of people, a higher prevalence of PTSD than of MDD was found-−22 vs. 14.3% in those who suffered the attack in childhood and 30.4 vs. 13% in those who suffered it in adolescence—. However, Maercker et al. (2004) found that women who had experienced a traumatic event during their adolescence were at higher risk of PTSD than of MDD, whereas women who had experienced a traumatic event during their childhood were at the same risk of PTSD as of MDD.

Perhaps the absence of significant differences or relationships between the age at which the attack occurred and the presence of emotional disorders or differential patterns of the presence of emotional disorders found in this study and discrepancies with the previous results of Maercker et al. (2004) have to do with the characteristics of the traumatic event in question. For example, people who experience prolonged or repetitive traumatic events during childhood or adolescence may present more emotional disorders as adults than people who experience such events as adults, but there are no differences in the age at which the traumatic event occurred when the event does not have these characteristics of chronicity and repetitiveness but instead is acute and isolated. In this sense, some data indicate the special resilience of children to some acute and isolated traumatic situations, which Bonanno and Diminich (2013) have called minimal-impact resilience, in contrast to resilience related to chronic and repetitive traumatic situations, which they have called emergent resilience. For example, in a sample of children with a mean age of 4 years who were followed-up for 3 years, Küenzlen et al. (2016) found that the children who had experienced an isolated traumatic event did not differ from the children who had not experienced traumatic events in the presence of behavioral problems over time, including emotional symptoms.

On another hand, the pattern of absence of differences or significant relationships may have to do with the level of analysis of the psychopathological consequences in adulthood; that is, it has to do with the fact that, in this study, only the presence of emotional disorders diagnosed according to the DSM-IV was examined. It could be expected that, compared to people who suffer terrorist attacks as adults, people who suffer terrorist attacks during childhood or adolescence would present higher levels of certain dimensions or continuous variables, broad or specific, related to mental health as adults. In this regard, Sarasua et al. (2012) found no differences between women who had suffered sexual assaults during childhood and those who had suffered them during adulthood in the presence of PTSD or emotional discomfort when they were evaluated as adults, but they did find that women who had suffered sexual assaults during childhood had more feelings of guilt.

Of course, both explanations for the pattern of the absence of differences or significant relationships are possible and complementary, and future research should try to replicate the results of this study and clarify which of these explanations is most appropriate or whether both are adequate, or whether other explanations are necessary or more accurate.

Finally, it is important to note that the findings of this study and the conclusions and explanations proposed should be valued taking into account the limitations of the study. The first is that we used a cross-sectional design when a longitudinal design would have been more desirable. The second is that the number of participants who had suffered an attack in childhood and adolescence was relatively small (*n* = 50 and 46, respectively), which could have compromised the statistical power of the study, especially concerning less frequent emotional disorders.

Despite these limitations, the results of this study are very robust when it comes to replicating very solid findings in the scientific literature. For example, all the regression analyses revealed that sex (or gender) was a variable significantly associated with the presence of the different emotional disorders (see Table 5), such that the prevalence of PTSD, MDD or anxiety disorders was higher in female than male victims of terrorism. This result replicates the consistent finding that those disorders are also more common among women compared to men in the general population (Kessler et al., 2005), including the Spanish general population (Haro et al., 2006). Furthermore, it also replicates the findings of the meta-analysis by Lowell et al. (2018) indicating that female gender is a significant, notable risk factor for PTSD related to the 11 September 2001 terrorist attacks.

On the other hand, despite those limitations, the results of this study concerning the influence of the age at which a terrorist attack was suffered are relatively robust and were similar regardless of the age chosen to distinguish childhood from adolescence (10 vs. 13 years), of including in the analyses participants who were under the age of 3 when they suffered a terrorist attack, of only analyzing participants who had been injured in an attack (direct victims), or only analyzing participants who had suffered a terrorist attack as children or adolescents. More importantly, the results of this study represent a novel contribution to the knowledge of the psychological impact of terrorist attacks on children and adolescents and, in general, of traumatic situations, indicating the need to examine in greater depth the resilience, resistance, and recovery of children and adolescents and how such capacities develop throughout their childhood and adolescence.

## Data Availability Statement

The raw data supporting the conclusions of this article will be made available by the authors, without undue reservation.

## Ethics Statement

The studies involving human participants were reviewed and approved by The Ethics Committee of the Faculty of Psychology at the Complutense University of Madrid. The patients/participants provided their written informed consent to participate in this study.

## Author Contributions

SP, JS, and MG-V contributed to conception and design of the study. SP, RF, NM, BC, CG, RN, and PA collected the data and organized the database. JS and SP performed the statistical analysis and wrote the first draft of the manuscript. JS, SP, and MG-V wrote sections of the manuscript. All authors contributed to manuscript revision, read, and approved the submitted version.

## Conflict of Interest

The authors declare that the research was conducted in the absence of any commercial or financial relationships that could be construed as a potential conflict of interest.
