# Supplementary material for: Growing Up With Terrorism: The Age at Which a Terrorist Attack Was Suffered and Emotional Disorders in Adulthood
Source: Front Psychol. 2021 Jun 18;12:700845. doi: 10.3389/fpsyg.2021.700845 (PMC8249802; doi:10.3389/fpsyg.2021.700845)
Supplement: Supplementary file 1 [file Table_1.docx]

Supplementary Material

Table 6. Prevalence (%) of emotional disorders in victim groups based on their age when they suffered the terrorist attack (0-9 years, 10-17 years, or 18 years or older) and significant differences between the groups in that prevalence (results based on the total sample of victims including the 35 participants who had initially been excluded for having suffered the terrorist attack before the age of 3)

| Emotional disorder | 0-9 years  (*n* = 50) | 10-17 years  (*n* = 46) | ≥ 18 years  (*n* = 470) | *p* of χ^2^ |
| --- | --- | --- | --- | --- |
| Posttraumatic stress disorder | 17.6 | 30.4 | 26.2 | .175 |
| Major depressive disorder | 10.7 | 13.0 | 19.8 | .091 |
| Panic disorder | 10.7 | 15.2 | 12.6 | .757 |
| Specific phobia | 11.9 | 10.9 | 14.9 | .611 |
| Generalized anxiety disorder | 7.1 | 13.0 | 10.7 | .496 |
| Social phobia | 1.2 | 6.5 | 5.8 | .195 |
| Agoraphobia | 1.2 | 2.2 | 5.5 | .152 |
| Obsessive-compulsive disorder | 9.4_a_ | 0.0_a_ | 4.1_a_ | .028 |
| Any mood disorder | 11.8_a_ | 26.1_a, b_ | 24.7_b_ | .029 |
| Any anxiety disorder | 32.9 | 37.0 | 38.2 | .656 |
| Any emotional disorder | 38.1 | 47.8 | 52.1 | .058 |

*Note*. In the case of significant χ^2^ tests with *p* < .05, the percentages with the same subscripts do not differ significantly at *p* < .05.

Table 7. Results of the multiple binary logistic regression analyses on the presence of emotional disorders in the victims who had suffered the terrorist attack as children or adolescents

| Emotional disorders / Predictors | B | *p* | Exp(B): *OR* |
| --- | --- | --- | --- |
| Posttraumatic stress disorder |  |  |  |
| Sex | 0.201 | .674 | 1.22 |
| Current age | -0.105 | .534 | 0.90 |
| Injured | 0.733 | .354 | 2.08 |
| Relative of the deceased | 0.218 | .706 | 1.24 |
| Time since the attack | 0.006 | .665 | 1.01 |
| Age at the time of the attack | 0.109 | .541 | 1.11 |
| Major depressive disorder |  |  |  |
| Sex | 0.181 | .780 | 1.19 |
| Current age | 0.152 | .583 | 1.16 |
| Injured | -0.845 | .495 | 0.43 |
| Relative of the deceased | 0.369 | .612 | 1.44 |
| Time since the attack | -0.019 | .392 | 0.98 |
| Age at the time of the attack | -0.199 | .489 | 0.82 |
| Panic disorder |  |  |  |
| Sex | 0.438 | .479 | 1.55 |
| Current age | -0.351 | .629 | 0.70 |
| Injured | 0.059 | .953 | 1.06 |
| Relative of the deceased | -0.417 | .560 | 0.66 |
| Time since the attack | 0.029 | .634 | 1.02 |
| Age at the time of the attack | 0.328 | .654 | 1.39 |
| Specific phobia |  |  |  |
| Sex | -0.131 | .850 | 0.88 |
| Current age | 0.993 | .204 | 2.70 |
| Injured | 0.357 | .793 | 1.43 |
| Relative of the deceased | 0.869 | .344 | 2.38 |
| Time since the attack | -0.085 | .189 | 0.92 |
| Age at the time of the attack | -0.997 | .209 | 0.37 |
| Any mood disorder |  |  |  |
| Sex | 0.275 | .610 | 1.32 |
| Current age | 0.776 | .202 | 2.17 |
| Injured | -0.226 | .820 | 0.79 |
| Relative of the deceased | 0.660 | .322 | 1.93 |
| Time since the attack | -0.069 | .171 | 0.93 |
| Age at the time of the attack | -0.749 | .223 | 0.473 |
| Any anxiety disorder |  |  |  |
| **Sex** | **1.012** | **.025** | **2.75** |
| Current age | 0.156 | .575 | 1.17 |
| Injured | -0.241 | .762 | 0.78 |
| Relative of the deceased | -0.299 | .570 | 0.74 |
| Time since the attack | -0.014 | .547 | 0.99 |
| Age at the time of the attack | -0.170 | .551 | 0.84 |
| Any emotional disorder |  |  |  |
| Sex | 0.914 | .035 | 2.49 |
| Current age | 0.102 | .646 | 1.11 |
| Injured | 0.634 | .422 | 1.89 |
| Relative of the deceased | 0.014 | .979 | 1.01 |
| Time since the attack | -0.011 | .545 | 0.99 |
| Age at the time of the attack | -0.112 | .625 | 0.89 |

*Note*. Statistically significant predictors appear in bold.

Table 8. Results of the multiple binary logistic regression analyses on the presence of emotional disorders in the total sample of victims of terrorism including the 35 participants who had initially been excluded for having suffered the terrorist attack before the age of 3

| Emotional disorders / Predictors | B | *p* | Exp(B): *OR* |
| --- | --- | --- | --- |
| Posttraumatic stress disorder |  |  |  |
| **Sex** | **0.895** | **.001** | **2.44** |
| Current age | 0.015 | .607 | 1.01 |
| **Injured** | **1.471** | **.001** | **4.35** |
| Relative of the deceased | 0.496 | .087 | 1.64 |
| Time since the attack | -0.004 | .146 | 0.99 |
| Age at the time of the attack | -0.034 | .244 | 0.97 |
| Major depressive disorder |  |  |  |
| **Sex** | **1.160** | **.001** | **3.19** |
| Current age | 0.015 | .632 | 1.01 |
| **Injured** | **1.041** | **.001** | **2.83** |
| Relative of the deceased | 0.417 | .186 | 1.52 |
| Time since the attack | -0.003 | .334 | 0.99 |
| Age at the time of the attack | -0.019 | .552 | 0.98 |
| Panic disorder |  |  |  |
| **Sex** | **1.198** | **.001** | **3.31** |
| Current age | 0.003 | .931 | 1.00 |
| **Injured** | **0.801** | **.024** | **2.23** |
| Relative of the deceased | 0.275 | .432 | 1.32 |
| Time since the attack | -0.002 | .451 | 0.99 |
| Age at the time of the attack | -0.031 | .395 | 0.97 |
| Specific phobia |  |  |  |
| **Sex** | **0.547** | **.035** | **1.73** |
| Current age | -0.005 | .886 | 0.99 |
| Injured | 0.166 | .602 | 1.18 |
| Relative of the deceased | 0.262 | .414 | 1.29 |
| Time since the attack | -0.003 | .230 | 0.99 |
| Age at the time of the attack | -0.011 | .755 | 0.99 |
| Any mood disorder |  |  |  |
| **Sex** | **0.982** | **.001** | **2.67** |
| Current age | 0.011 | .702 | 1.01 |
| **Injured** | **0.998** | **.001** | **2.71** |
| **Relative of the deceased** | **0.576** | **.043** | **1.78** |
| Time since the attack | -0.002 | .344 | 0.99 |
| Age at the time of the attack | -0.018 | .540 | 0.98 |
| Any anxiety disorder |  |  |  |
| **Sex** | **0.933** | **.001** | **2.54** |
| Current age | 0.002 | .943 | 1.00 |
| **Injured** | **0.690** | **.005** | **1.99** |
| **Relative of the deceased** | **0.481** | **.044** | **1.62** |
| Time since the attack | -0.004 | .131 | 0.99 |
| Age at the time of the attack | -0.025 | .316 | 0.97 |
| Any emotional disorder |  |  |  |
| **Sex** | **1.041** | **.001** | **2.83** |
| Current age | 0.006 | .833 | 1.01 |
| **Injured** | **1.113** | **.001** | **3.04** |
| **Relative of the deceased** | **0.511** | **.028** | **1.67** |
| Time since the attack | -0.003 | .152 | 0.99 |
| Age at the time of the attack | -0.023 | .415 | 0.98 |

*Note*. Statistically significant predictors appear in bold.
